# Age, socioeconomic status, and weight status as determinants of dietary patterns among German youth: findings from the LIFE child study

**DOI:** 10.3389/fnut.2025.1578176

**Published:** 2025-04-30

**Authors:** Emmelie Hähnel, Carolin Sobek, Peggy Ober, Wieland Kiess, Mandy Vogel

**Affiliations:** ^1^LIFE Child, Medical Faculty, Hospital for Children and Adolescents, Leipzig University, Leipzig, Germany; ^2^Center for Pediatric Research (CPL), Medical Faculty, Hospital for Children and Adolescents, Leipzig University, Leipzig, Germany; ^3^German Center for Child and Adolescent Health (DZKJ), Leipzig/Dresden Partner Site, Leipzig, Germany

**Keywords:** nutrition, dietary patterns, children, adolescents, socioeconomic status, overweight, obesity, sex

## Abstract

**Background:**

Malnutrition and its consequences, such as obesity, are growing problems, especially in disadvantaged subpopulations. In order to pinpoint possible contributors to children’s nutritional habits, we examined potential determinants as age, sex, socioeconomic status, and weight status of different dietary patterns (dp) in a large German research project.

**Methods:**

The data was collected within a population-based longitudinal cohort study. We used the Food Frequency Questionnaire (FFQ) to assess food intake in 484 children and adolescents aged 5–18 years across 1,068 visits. Cluster analysis was used to identify food groups. Study participants who consumed food groups with a similar frequency were grouped together as dietary patterns. We applied logistic and linear regression to test for whether group membership in different food groups and dietary patterns was associated with age, sex, socioeconomic status (SES), or body mass index (BMI).

**Results:**

Overall, food consumption frequency decreased with age, including healthy foods like fruits (*β* = −0.39, *p* < 0.001) and vegetables (*β* = −0.17, *p* = 0.020). Boys consumed more meat and carbohydrates, milk/egg products, and junk food than girls did, but dietary patterns showed no significant sex differences. There was a trend toward a healthier diet with increasing SES (OR = 1.33, *p* < 0.001). Children with overweight or obesity were less likely to follow an infrequent diet (OR_OW_ = 0.56, *p* = 0.075; OR_OB_ = 0.41, *p* < 0.001) and were not significantly underrepresented in the healthiest pattern but were more likely to follow a neutral diet (OR_OW_ = 4.14, *p* = 0.042; OR_OB_ = 1.47, *p* = 0.504).

**Conclusion:**

Our study identified age and SES as key factors in children’s and adolescents’ nutrition, highlighting their importance for improvement measures. The findings on weight and diet suggest both the complexity of obesity aetiology and potential reporting bias in certain weight groups.

## 1 Introduction

A healthy and balanced diet is essential for a child’s growth and development (1, 2). Additionally, dietary patterns learned in childhood often persist into adulthood (3, 4). Thus, establishing a well-balanced diet early in childhood significantly lowers the risk for short-and long-term health problems, for example, dental disease, overweight, cardiovascular disease, diabetes mellitus, cancer, and osteoporosis (5).

Even though multiple studies have reported a partial improvement in the quality of children’s and adolescents’ diets during the last decade, the quality is still low. Dietary recommendations for various foods and nutrients are often not fulfilled. Children exceed the limit for daily sugar intake, but they do not meet the recommendations for fruits and vegetables (6–8).

The World Health Organization (WHO) describes obesity as one of the biggest health challenges of the 21st century (9) - independent of socioeconomic status (SES) (10). In 2022, the WHO estimated that 390 million (20%) children and adolescents between 5 and 19 years of age were overweight. Of these, 160 million (8%) were obese (11). In Germany, the percentage of children with overweight was approximately 15.4% (2014–2017) (12). Overweight and obesity are linked to an increased risk of physical and psychological illnesses (13, 14). Moreover, childhood obesity and related health risks can persist into adulthood (13, 15, 16), thereby increasing the burden on society through rising medical care costs and lower work productivity (17). A recent study even identified an excessive body mass index (BMI) during childhood as a risk for cardiovascular disease in adulthood irrespective of adult weight status in a pooled cohort of more than 10,000 subjects (16).

The causes of the increasing obesity rates are multifactorial. High-calorie nutrition, lack of exercise, and high media use play major roles. Furthermore, the consumption of convenience foods, snacking between meals, and portion sizes are increasing. Overall, these trends lead to energy intake that exceeds energy expenditure and results in weight gain (1).

Germany is one of the wealthiest countries in the world and has a good social security system. Nevertheless, even within Germany, significant variability in living conditions contributes to significant variability in how children are brought up (18). Also worldwide, 333 million children live in extreme poverty (19), and not only low-income countries are affected. 25% of children in the EU are living at risk of poverty (20), and 11 million children (15%) in the US are living in poverty (21). Unfortunately, children’s social backgrounds are tightly linked to different dimensions of child health (22), and nutrition is one of them. Widespread socioeconomic inequalities in dietary behavior contribute to nutrition-related health inequalities, and, as mentioned above, because unfavorable nutrition behaviors that are manifested in childhood often persist into adulthood and determine long-term health outcomes (23), such behaviors can contribute to the transgenerational transmission of health inequalities.

Indeed, the gap is widening, especially in Germany and other wealthy countries, due to rising income inequalities within countries (24). As a result, more disadvantaged families have fewer opportunities to consume a balanced diet; tend to consume fewer fruits and vegetables and more fatty foods; have social, cultural, and health restrictions; have a smaller choice of food; and cannot use food as a medium for individuation and community building (25).

The diets and nutritional behaviors of children and adolescents are shaped by numerous factors, which have been studied to varying extents. Research has shown that the quality of children’s diets changes with age (26, 27), that girls and boys may differ in their diets (7), and that socially disadvantaged families often turn to cheaper, less nutrient-dense foods (28). Moreover, a healthy diet has been associated with a lower BMI (27). However, the precise role of these factors and how they interact is still not fully understood, and many studies have only examined individual factors in isolation. This study builds on previous work by examining the combined effects of age, sex, socioeconomic status, and BMI on dietary habits. Unlike previous research, we focus not just on individual foods or food groups, but on broader dietary patterns. A dietary pattern refers to the overall combination and frequency of foods and beverages habitually consumed over time, reflecting the quality and balance of an individual’s or population’s diet. It captures the interactions between different dietary components rather than focusing on individual nutrients or foods. Through the dietary patterns identified in our research, we aim to create a more nuanced classification of eating habits that transcends the simplistic “healthy” versus “unhealthy” dichotomy. Additionally, we include both a healthy cohort and an obesity cohort to compare the dietary habits of healthy and obese children, offering deeper insights into the eating patterns of both groups. The current study is aimed at addressing the following research questions: (1) what dietary patterns can be identified for children and adolescents in the LIFE Child population? (2) What are the associations between age, sex, BMI, and SES and children’s/adolescents’ food intake or dietary patterns?

## 2 Materials and methods

### 2.1 Study design and population

The LIFE Child study is an ongoing population-based longitudinal cohort study conducted by the Research Center for Civilization Diseases in Leipzig (Central Germany). The study began in 2011. It comprises three cohorts (birth/health/obesity) and aims to capture individual development through annual follow-ups. A multi-professional team collects a wide range of data, including physical examinations, interviews, questionnaires, biological samples, and standardized tests. The study is described in detail elsewhere (29, 30).

In this cross-sectional analysis, we included all children and adolescents from the health and obesity cohorts between the ages of 5 and 18 years who completed the Food Frequency Questionnaire (FFQ) between 2011 and 2017.

The study meets the criteria set forth by the Declaration of Helsinki. The Ethics Committee of the Medical Faculty of Leipzig University (Reg. no. 477/19-ek) approved the study. The Ethics Committee is registered as an Institutional Review Board with the Office for Human Research Protection (IORG0001320 and IRB00001750). All children over 12 years of age and their parents gave written consent before the children were included in the study.

### 2.2 Data

The FFQ captures the mean consumption frequencies and portion sizes (per week) of different foods consumed within the last year. This FFQ version assessed 94 different foods, drinks, and supplements. Up to the age of 12 years, the questionnaire was filled out by a parent along with their child in the study center. Older children and adolescents answered the questions on their own. Participants could choose between “never,” “1 time in 4 weeks,” “2–3 times in 4 weeks,” “1 time per week,” “2–3 times per week,” “4–6 times per week,” “1 time per day,” “2–3 times per day,” and “4 times per day or more.” A hierarchical cluster analysis was performed based on the information on the consumption frequencies of the individual foods in the FFQ. This resulted in food groups (fg) containing foods that were consumed with similar frequency by the children. A second hierarchical cluster analysis was applied to these food groups, from which dietary patterns (dp) emerged. These consisted of children who consumed the respective food groups with similar frequency.

All subjects were included in the analysis who completed the questionnaire at least once and who had at least 40 non-missing values when answering the questionnaire. This was the case for almost all participants, leading to a very low rate of missing values overall.

The SES was assessed as a composite score combining information on the equalized household net income, parents’ education, and occupational status, as defined by Winkler et al. (31). Each sub-score ranged from 1 to 7 points, resulting in a total range of 3–21 points. A total score between 3 and 8 points was defined as low SES, between 9 and 14 points as medium SES, and between 15 and 21 points as high SES (31).

Height and weight were measured in light underwear by trained staff. BMI was calculated as the ratio of body weight to the square of body height. Using the current S3-guideline of the Working Group on Childhood and Adolescent Obesity (AGA) of the German Obesity Society (DAG) and the German Society of Pediatrics and Adolescent Medicine (DGKJ) (32), the BMI values were transformed into sex-and age-adjusted standard deviation scores (SDSs). Subsequently, SDS values ≥ −1.28 but <1.28 were categorized as normal weight, between 1.28 and 1.88 as overweight, and above 1.88 as obese (33).

### 2.3 Statistical analysis

To minimize memory errors and social desirability, we paid attention to the following things:

Each participant was guaranteed anonymity when completing the questionnaire and the questions were formulated in a neutral way. Foods that are difficult for children or lay people to distinguish were grouped together: Freshwater fish, saltwater fish, and herring were summarized as “fish,” various kinds of cake as “cake,” and butter and margarine as well as different nuts and seeds were grouped together. Furthermore, foods that were difficult to identify as ingredients within the dishes, especially for children (e.g., cream, buttermilk, fats, or fat content) were excluded. Additionally, foods that were consumed by very few participants were not included (e.g., sugar beet or innards). Due to the insufficient validity of the reported portion sizes, only consumption frequencies were considered (34). The participants were asked to provide information on their eating habits over the course of a year. This allows seasonal distortions to be mitigated. We decided against analysing beverages, as this information is very susceptible to errors in questionnaires. In addition, the questions were too imprecise to draw concrete conclusions regarding the nutrient composition and thus quality. Ice cream was excluded, as this is a product that is mainly consumed seasonally and therefore the frequency of consumption varies greatly. Dietary supplements were also not included in the analyses, due to a large amount of missing data and the unclear assessment of the influence of dietary supplements on the nutritional quality of the participants. In addition, our focus was on a natural diet.

The statistical analyses were performed in “R” (version 4.4.1) (35). First, using hierarchical cluster analysis, foods were clustered according to similar consumption frequencies. Subsequently, a second hierarchical cluster analysis was performed to identify the different dietary patterns among children and adolescents. The associations between group memberships, age, sex, BMI, and SES were examined using multiple linear and logistic regression. We accounted for multiple measurements per participant by including a random intercept in the models and checked for interactions between predictors. Effects are reported as slopes/differences and odds ratios (OR), including 95% confidence intervals. The significance level was set to *α* = 0.05.

## 3 Results

### 3.1 Cohort description

In total, the FFQ was completed 1.068 times by 484 participants (49.6% female; 50.4% male). Table 1 presents the basic sample characteristics at the first visit. The age classification is based on whether they completed the FFQ themselves (≥ 13 years) or along with a parent (< 13 years).

**Table 1:** Characterization of the study sample.

	Study population (*n* = 484)	Female (*n* = 240)	Male (*n* = 244)	Interaction with sex	Total
	*n* (%)/mean ± SD			*p*-value	*N*
Age (years)	11.8 (2.81)	12.0 (3.01)	11.7 (2.61)	0.287	484
Age groups				0.170	484
< 13 years	314 (64.9%)	148 (61.7%)	166 (68.0%)		
≥ 13 years	170 (35.1%)	92 (38.3%)	78 (32.0%)		
Number of visits	2.21 (1.22)	2.23 (1.22)	2.18 (1.23)	0.634	484
SES				0.363	447
High	82 (18.3%)	37 (17.1%)	45 (19.6%)		
Medium	276 (61.7%)	131 (60.4%)	145 (63.0%)		
Low	89 (19.9%)	49 (22.6%)	40 (17.4%)		
BMI (kg/m^2^)	23.4 (6.51)	23.6 (6.63)	23.2 (6.40)	0.451	481
BMI-SDS	1.16 (1.41)	1.18 (1.49)	1.14 (1.33)	0.787	481
Weight groups				0.461	481
Underweight	18 (3.74%)	12 (5.02%)	6 (2.48%)		
Normal weight	209 (43.5%)	99 (41.4%)	110 (45.5%)		
Overweight	46 (9.56%)	23 (9.62%)	23 (9.50%)		
Obesity	208 (43.2%)	105 (43.9%)	103 (42.6%)		
Dietary patterns				0.235	484
Mixed 1 dp	106 (21.9%)	55 (22.9%)	51 (20.9%)		
Mixed 2 dp	61 (12.6%)	27 (11.2%)	34 (13.9%)		
Non-frequent dp	174 (36.0%)	87 (36.2%)	87 (35.7%)		
Balanced dp	67 (13.8%)	27 (11.2%)	40 (16.4%)		
Bread dp	66 (13.6%)	40 (16.7%)	26 (10.7%)		
Frequent dp	10 (2.07%)	4 (1.67%)	6 (2.46%)		

Continuous variables are given as means and standard deviations, and categorial variables are given as counts and percentages. n, count; N, total; SES, socioeconomic status; BMI, body mass index; SDS, standard deviation score; dp, dietary pattern.

### 3.2 Food groups (fg)

**Figure 1:**
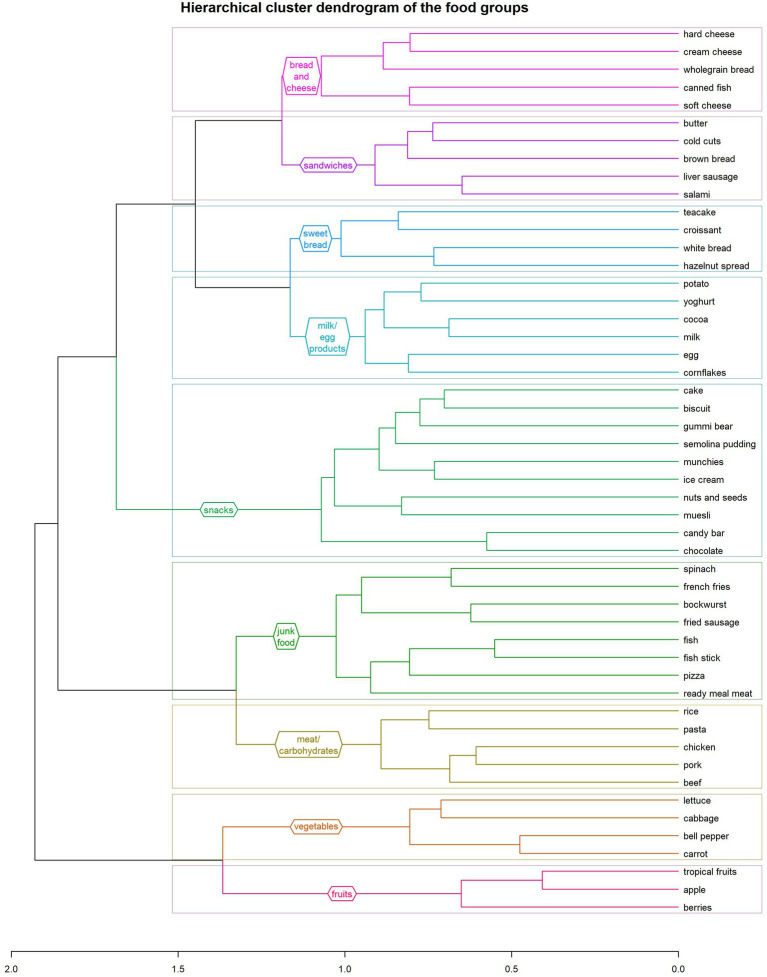
Hierarchical cluster dendrogram of the nine food groups identified in this study: (1) fruits, (2) vegetables, (3) meat and carbohydrates, (4) junk food, (5) snacks, (6) milk/egg products, (7) sweet bread 2, (8) sandwiches, (9) bread and cheese.

Nine food groups with similar consumption frequencies were identified and are shown in detail in Figure 1.

### 3.3 Dietary patterns (dp)

**Figure 2:**
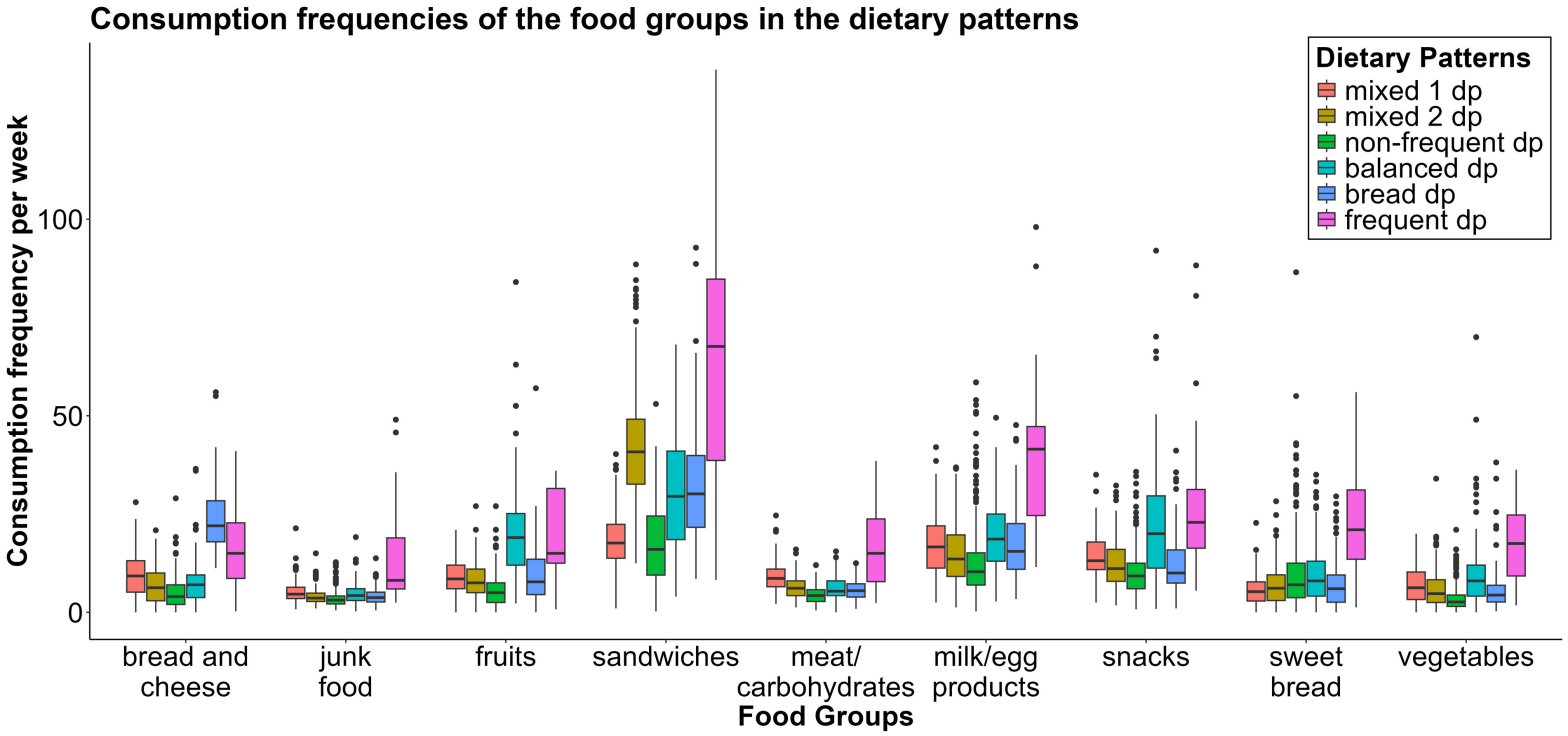
Boxplot showing the distribution of the consumption frequencies per week of the nine food groups between the six dietary patterns. dp, dietary pattern.

On the basis of the food groups, we identified six different dietary patterns (Figure 2). Across all the patterns, the food group that was consumed most frequently was the*“sandwiches fg,”* and the one consumed least frequently was the *“junk food fg.”* Also, for every dietary pattern, children and adolescents ate the*“fruits fg”* more often than the *“vegetables fg.”* Based on the consumption frequency of the various food groups within the dietary patterns, we have categorized and named them. *“Mixed 1 dp”* and *“mixed 2 dp”* show a similar distribution of consumption frequencies of the food groups and, in comparison with the other dietary patterns, include all food groups with a nearly similar frequency. Compared with the other dietary patterns, subjects who adhered to the *“mixed 1dp”* consumed the *“sweet bread fg”* least often and the *“junk food fg”* and the *“meat and carbohydrates fg”* more frequently. The *“mixed 2 dp”* showed the second-highest consumption frequency of the *“sandwiches fg”* out of all the dietary patterns, which is also the main difference between the two mixed dietary patterns.

The *“non-frequent dp”* ate all the food groups least often of all, except the *“sweet bread fg.”*

The *“balanced dp”* consumed the *“fruits fg”* more frequently than all the other dietary patterns did. They also ate the *“vegetables fg,”* the *“milk/egg products fg,”* the *“sweet bread fg,”* and the *“snacks fg”* more often than the other dietary pattern groups did. This pattern showed also a mixed ratio of consumption frequencies, but due to the higher frequencies of healthy food groups, this group was designated as a balanced diet.

The *“bread dp”* consumed the *“bread and cheese fg”* more frequently than the other patterns did. The last dietary pattern *(“frequent dp”)* consumed all the food groups except the *“fruits fg”* and the *“bread and cheese fg”* more frequently than all the other dietary patterns did. Supplementary Figures 1–3 show the consumption frequencies for the nine food groups across the six dietary patterns in a box plot (Supplementary Figure 1) and polar plots (Supplementary Figures 2, 3).

The largest group of children and adolescents adhered to the *“non-frequent dp”* (36.0%), followed by the *“mixed 1 dp”* (21.9%). The distributions across the *“mixed 2 dp”* (12.6%), *“balanced dp”* (13.8%), and *“bread dp”* (13.6%) were almost equal (Table 1). The *“frequent dp,”* with only 10 participants (2.1%), was not included in any further analyses.

### 3.4 Correlates of food groups

Supplementary Table 1 in the supplement presents a summary of all the results.

#### 3.4.1 Age and sex

**Figure 3:**
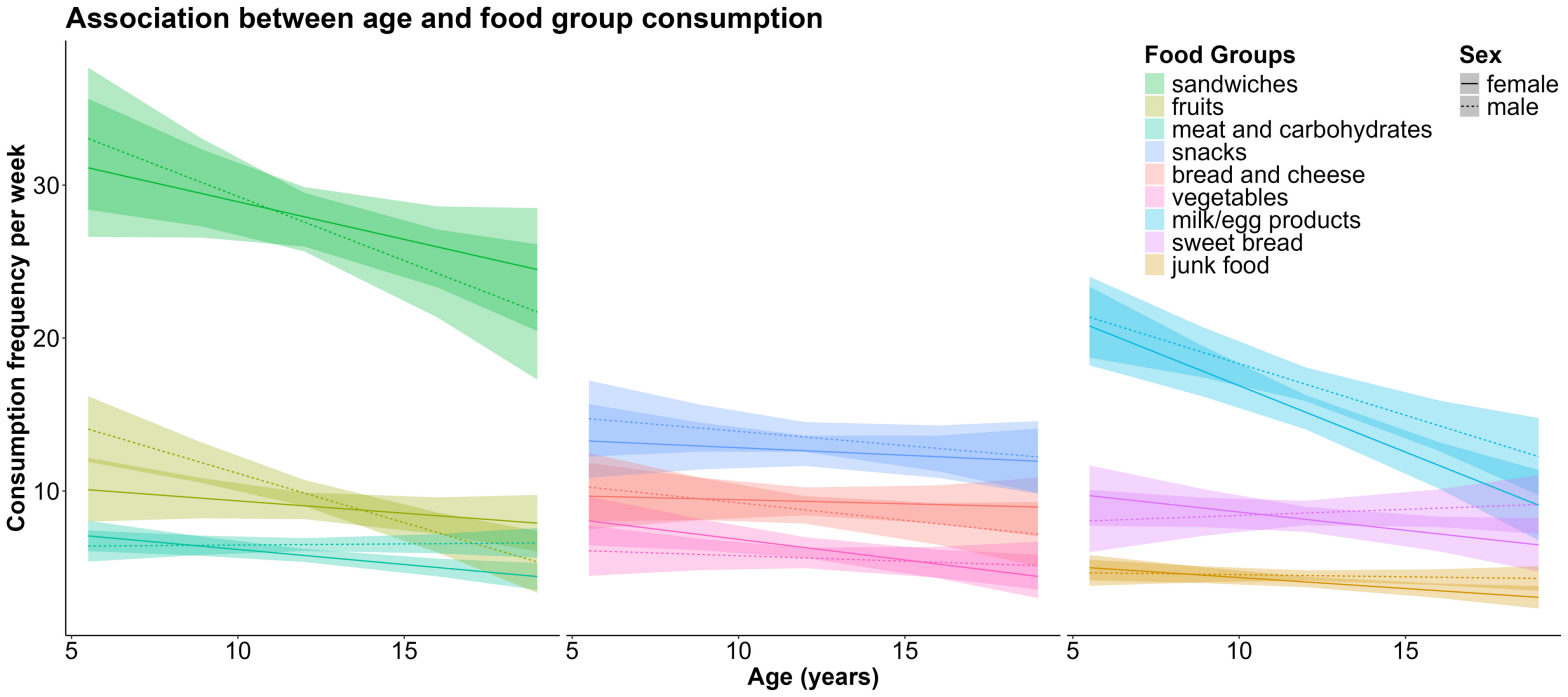
Associations between age, sex and weekly consumption of the nine food groups.

As age increased, the consumption of the following food groups decreased: *“fruits fg”* [*β* = −0.39, CI = −0.58 – (−0.20), *p* < 0.001], *“vegetables fg”* [*β* = −0.17, CI = −0.32 – (−0.03), *p* = 0.020], *“meat and carbohydrates fg”* [*β* = −0.11, CI = −0.20 – (−0.01), *p* = 0.025], *“junk food fg”* [*β* = −0.09, CI = −0.17 – (−0.02), *p* = 0.017], *“milk/egg products fg”* [*β* = −0.79, CI = −1.03 – (−0.56), *p* < 0.001], and *“sandwiches fg”* [*β* = −0.65, CI = −1.06 – (−0.24), *p* = 0.002]. The remaining food groups were also eaten less frequently by older children, but the trends were not statistically significant (Figure 3).

**Figure 4:**
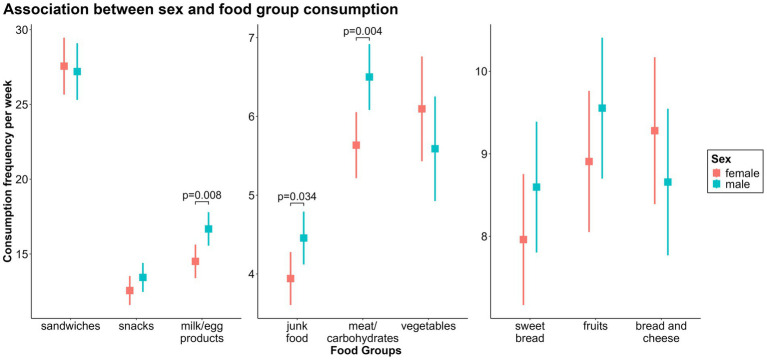
Associations between sex and weekly consumption of the nine food groups.

Boys consumed the following food groups more frequently than girls did: *“meat and carbohydrates fg”* (*β* = 0.87, CI = 0.27–1.46, *p* = 0.004), *“junk food fg”* (*β* = 0.51, CI = 0.04–0.99, *p* = 0.034), and *“milk/egg products fg”* (*β* = 2.17, CI = 0.58–3.75, *p* = 0.008). There were no significant sex differences in the consumption of the remaining food groups (Figure 4).

#### 3.4.2 Socioeconomic status (SES)

Children from families with medium [*β* = −0.93, CI = −1.45 – (−0.42), *p* < 0.001] or high SES [*β* = −0.95, CI = −1.62 – (−0.29), *p* = 0.005] consumed the*“junk food fg”* less frequently than children with low SES.

A similar association, consistent in direction and with an even stronger effect, was found between SES and the consumption frequency of the *“sandwiches fg”* [*β*_SES medium_ = −4.50, CI = −7.42 – (−1.57), *p* = 0.003; *β*_SES high_ = −7.86, CI = −11.6 – (−4.08), *p* < 0.001].

The consumption frequency of the *“snacks fg”* had an inverse association. Children with medium (*β* = 1.76, CI = 0.23–3.29, *p* = 0.024) or high SES (*β* = 2.69, CI = 0.74–4.65, *p* = 0.007) consumed the *“snacks fg”* more frequently than children from families with low SES.

**Figure 5:**
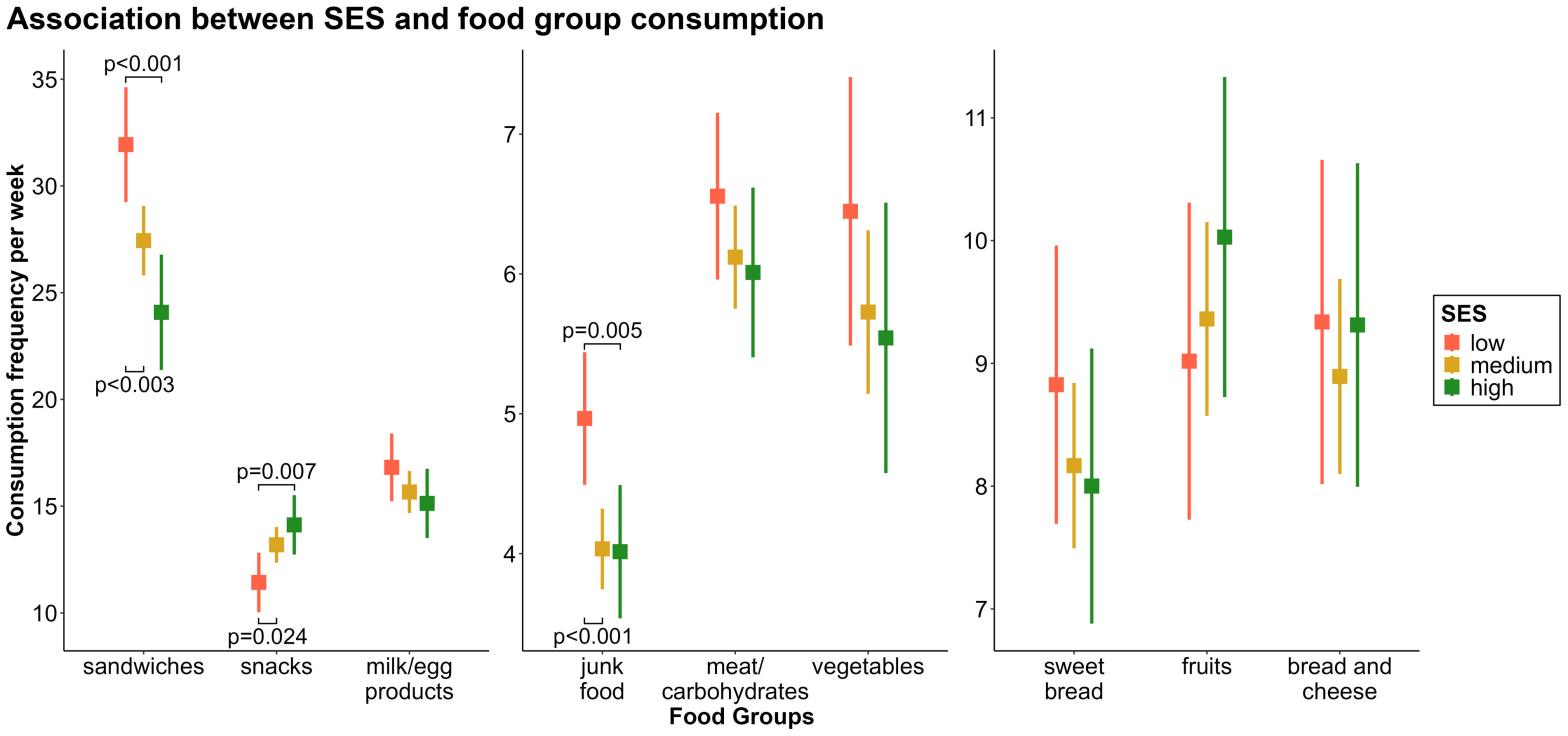
Associations between socioeconomic status (SES) and weekly consumption of the nine food groups.

There were no further significant associations between SES and the consumption frequencies of the remaining food groups (Figure 5).

#### 3.4.3 Weight groups

Children with overweight [*β* = −2.78, CI = −4.81 – (−0.75), *p* = 0.007] or obesity [*β* = −3.42, CI = −4.85 – (−1.99), *p* < 0.001] consumed the *“snacks fg”* significantly less frequently than children with normal weight.

Similar results were found when comparing the consumption frequency of the *“sweet bread fg”* between children with overweight [*β* = −2.23, CI = −3.90 – (−0.57), *p* = 0.008] or obesity [*β* = −2.36, CI = −3.53 – (−1.18), *p* < 0.001] and children with normal weight.

**Figure 6:**
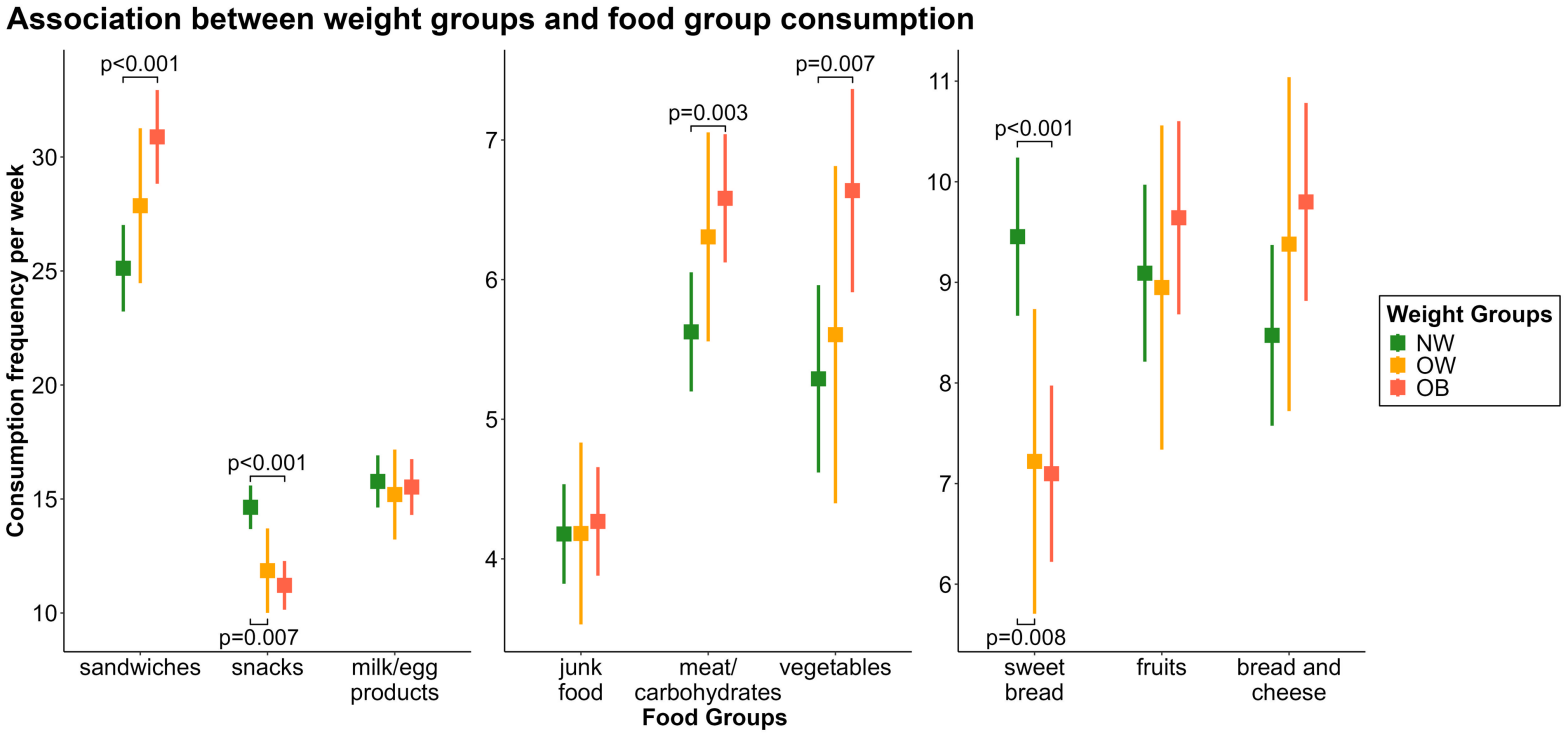
Associations between the weight groups and weekly consumption of the nine food groups. OB, obese; OW, overweight; NW, normal weight.

Children with obesity consumed the *“meat and carbohydrates fg”* (*β* = 0.96, CI = 0.33–1.58, *p* = 0.003), *“vegetables fg”* (*β* = 1.35, CI = 0.36–2.33, *p* = 0.007), and *“sandwiches fg”* (*β* = 5.76, CI = 2.98–8.54, *p* < 0.001) significantly more frequently than children with normal weight. For children with overweight, the effects had the same direction but smaller effect sizes, which were not statistically significant. The consumption frequencies of the remaining food groups did not show any significant associations (Figure 6).

### 3.5 Correlates of dietary patterns

**Figure 7:**
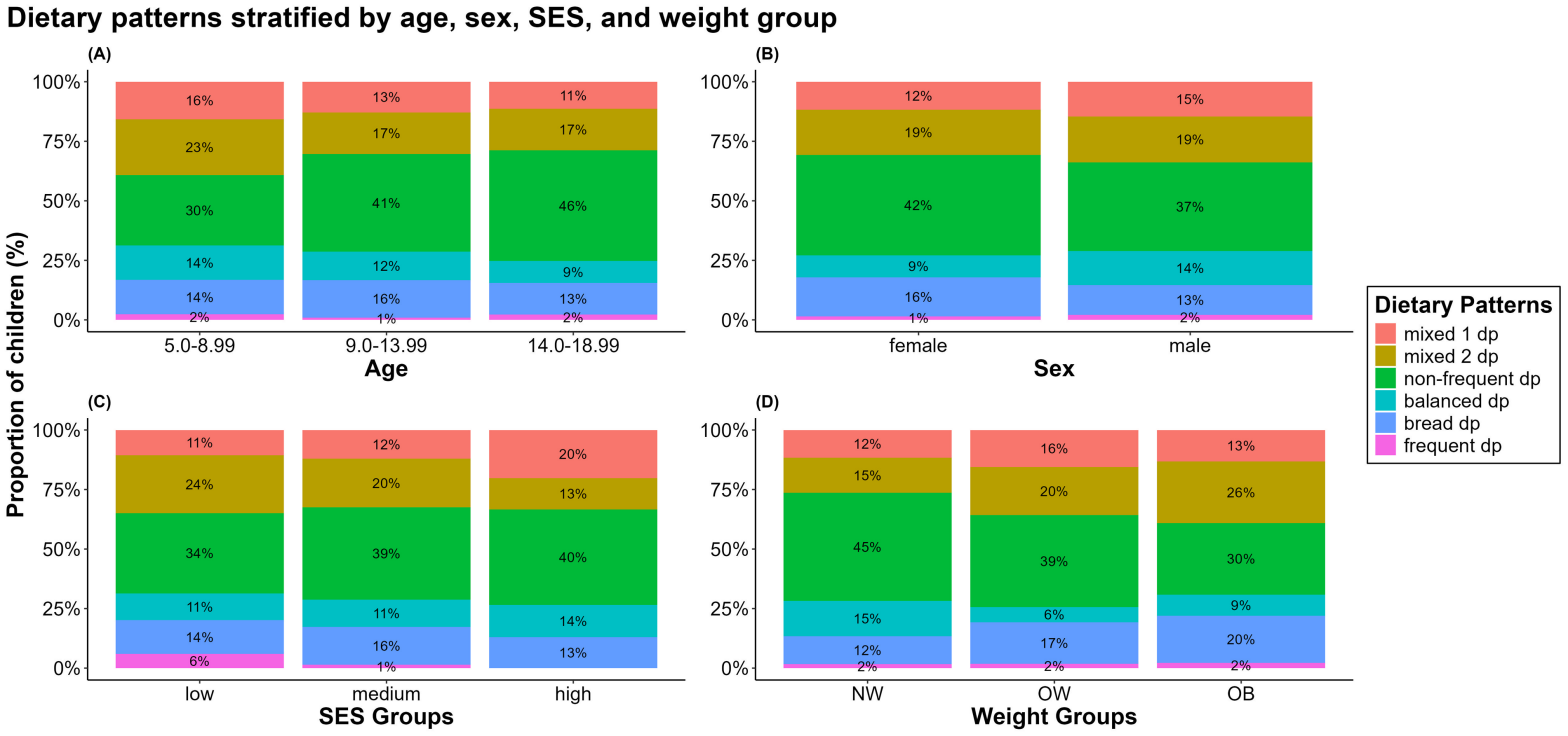
Distributions of dietary patterns stratified by **(A)** age groups, **(B)** sex, **(C)** SES groups, and **(D)** weight groups. Primary school age (5.50–10.99 years), early puberty (11.00–13.99 years), late puberty (14.00–18.99 years). NW, normal weight; OW, overweight; OB, obese.

Supplementary Table 2 in the supplement presents an overview of all the results. Figure 7 presents the frequency distributions for each predictor stratified by dietary pattern. We also tested for interactions for all predictors but did not find any significant results.

#### 3.5.1 Age and sex

With increasing age, the proportion of children and adolescents with the *“non-frequent dp”* increased (OR = 1.16, CI = 1.08–1.25, *p* < 0.001), whereas the proportions with the *“mixed 2 dp”* (OR = 0.92, CI = 0.86–0.98, *p* = 0.014) and *“balanced dp”* (OR = 0.85, CI = 0.72–0.996, *p* = 0.045) decreased. We found no statistically significant differences in the dietary patterns between boys and girls, only trends.

#### 3.5.2 Socioeconomic status (SES)

Children with high SES were significantly more likely to be in the *“mixed 1 dp”* than those with low (OR = 2.90, CI = 1.13–7.44, *p* = 0.027) or medium SES were (OR = 2.35, CI = 1.16–4.77, *p* = 0.018). The difference between the medium and low SES groups was not statistically significant (OR = 1.23, CI = 0.57–2.68, *p* = 0.598).

We found the opposite trend for the *“mixed 2 dp”* such that children with high SES were less likely to be in this group than those with low (OR = 0.44, CI = 0.24–0.81, *p* = 0.008) or medium SES were (OR = 0.57, CI = 0.35–0.95, *p* = 0.030). Again, the difference between the medium and low SES groups was not statistically significant (OR = 0.77, CI = 0.48–1.22, *p* = 0.270).

In contrast to children with low (OR = 1.14, CI = 0.90–1.43, *p* = 0.195) or medium (OR = 1.33, CI = 1.10–1.62, *p* < 0.001) SES, children with high SES were more likely to belong to the *“balanced dp.”* Although the effect sizes were similar, the effect was significant only for medium SES but not for low SES. Children with medium SES were less likely to be in this pattern group than those with low SES (OR = 0.85, CI = 0.70–1.03, *p* = 0.049). The effect was significant, but weak. We did not find significant associations between socioeconomic status and the remaining patterns.

#### 3.5.3 Weight groups

Children with overweight or obesity were more likely to follow the *“mixed 1 dp”* than their normal weight counterparts were. The effect was stronger and statistically significant only for children with overweight (OR_OW_ = 4.14, CI = 1.05–16.3, *p* = 0.042; OR_OB_ = 1.47, CI = 0.48–4.51, *p* = 0.504).

Similar but weaker effects were found for the *“mixed 2 dp,”* this time reaching statistical significance only for children with obesity (OR_OW_ = 1.49, CI = 0.83–2.70, *p* = 0.186; OR_OB_ = 2.14, CI = 1.45–3.15, *p* < 0.001).

We found effects in the same direction for the *“bread dp”* with an OR of 1.76 (CI = 0.80–3.90, *p* = 0.161) for children with overweight and an OR of 1.98 (CI = 1.14–3.44, *p* = 0.015) for children with obesity. Again, the result was statistically significant only for children with obesity.

The opposite trend was found for the *“non-frequent dp”* (OR_OW_ = 0.56, CI = 0.30–1.06, *p* = 0.075; OR_OB_ = 0.41, CI = 0.26–0.64, *p* < 0.001) and *“balanced dp”* (OR_OW_ = 0.32, CI = 0.07–1.52, *p* = 0.150; OR_OB_ = 0.60, CI = 0.19–1.89, *p* = 0.379). Although these two effects had the same direction, the effect was not statistically significant in the *“balanced dp.”* In addition, the effect in the *“balanced dp”* was weaker for children with obesity than for children with overweight.

## 4 Discussion

### 4.1 Summary of the main results

The aim of our study was to use FFQ data to investigate how certain dietary patterns in food consumption were associated with age, sex, SES, and BMI-SDS in 484 children and adolescents. First, we identified food groups and groups with highly discriminating dietary patterns, and second, we computed associations between the food groups or dietary patterns and variables that were hypothesized to be predictors of these. We identified nine food groups and six dietary patterns. The dietary patterns were significantly associated with age, SES, and the weight groups but not with sex. On the other hand, we found associations between food group consumption frequencies and all four predictors.

### 4.2 Gradation of nutritional quality

First, the food groups and dietary patterns could be categorized as unhealthy, neutral, and healthy. The German Nutrition Society (DGE) (36) recommends a primarily plant based diet with adequate intake of fruits, vegetables, nuts, and fiber. This diet should be supplemented with limited amounts of animal products such as milk, eggs, fish, and meat, whereas processed, high-fat, sugary, and high-salt foods should be minimized. Thus, we could classify our food groups as given in Table 2.

**Table 2:** Classification of food groups on the basis of nutritional quality.

Healthy	Neutral	Unhealthy
Fruits	Meat and carbohydrates	Junk food
Vegetables	Milk/egg products	Snacks
Bread and cheese		Sweet bread
		Sandwiches

*“Mixed 1 dp”* and *“mixed 2 dp”* are similar dietary patterns, except for a higher frequency of the consumption of the *“sandwiches fg”* in the *“mixed 2 dp.”* Both patterns contain a mix of food groups of all diet qualities and are therefore considered neutral.

*“Non-frequent dp”* is characterized by the least frequent consumption of nearly all the food groups compared with the other patterns, suggesting lower meal frequencies overall. Studies indicate that higher meal frequencies are associated with better diet quality (37–39). Thus, due to its low frequency of consumption of any food groups and high proportion of unhealthy food groups, this pattern is categorized as unhealthy.

Compared with the other patterns, the *“balanced dp”* shows higher over-all frequencies of the consumption of all food groups, especially the healthy ones. Thus, this pattern is classified as healthy.

*“Bread dp”* features a high frequency of the consumption of the *“bread and cheese fg”* and *“sandwiches fg,”* with an average consumption frequency of other food groups, rendering it neutral in dietary quality.

### 4.3 Nutrition and examined predictors

#### 4.3.1 Age and sex

We found lower consumption frequencies of the *“fruits fg,” “vegetables fg,” “meat and carbohydrates fg,” “milk/egg products fg,” “junk food fg,”* and *“sandwiches fg”* with increasing age. Furthermore, adolescents were more likely to adhere to the *“non-frequent dp”* than younger children. This result is plausible because older children tend to eat less frequently but in larger quantities and therefore tend to consume more overall (40–42). Additionally, research has shown that skipping breakfast is more common among older than younger children (43), a tendency that thereby has the potential to contribute to a lower consumption frequency.

Body image and satisfaction also become more significant factors as children grow older, leading adolescents to skip meals to achieve body ideals. Hulbert et al. (44) highlighted this development in the Health Behavior in School-Aged Children (HBSC) England national report in 2023, noting that satisfaction with body size and image decreases with age. Adolescents often use methods to regulate their food intake, alongside increased exercise, to reach body ideals (44). This tendency could also contribute to the increasing proportion of children in the *“non-frequent dp*” with age.

Some studies have reported trends that go counter to our results for fruits and vegetables (40, 45, 46), whereas others have found similar trends (42, 47). Demory-Luce et al. (42) observed a decrease in the consumption of vegetables, fruits, meat, and milk with increasing age. They also reported a decrease in dietary quality from childhood to young adulthood, manifesting in a 10% reduction in the total consumption (in grams) of nutrient-dense foods as participants aged (42). This finding is in line with our finding that adolescents were less likely to follow the healthier *“balanced dp.”* This can be partly explained by the growing independence from parents and the increasing influence of friends. Young children are entirely dependent on their parents, but as they age, their dependence on them decreases while peer influence rises (48). Belonging to a group shapes preferences, decisions, and attitudes, with adolescents often adopting eating habits similar to their friends. If their peers eat unhealthily, they are more likely to do the same (49). On the other hand, media consumption and exposure to advertising also play a significant role. As children grow older, their media consumption increases, making them particularly vulnerable. Advertising is pervasive, affecting thoughts, knowledge, behavior, attitudes, and consumption patterns, especially with the promotion of cheap, energy-dense, and visually appealing foods (50). This has been linked to higher consumption of snacks and sugar, which could possibly contribute to obesity in children (51). Efforts to reduce unhealthy food consumption through limiting advertising, particularly on TV and social media, are underway, though full implementation remains a challenge (52, 53).

When looking at specific food groups, boys consumed the *“meat and carbohydrates fg,” “junk food fg,”* and *“milk/egg products fg”* more frequently than girls did. Various studies have identified similar sex-related differences in the consumption of meat, milk, and egg products when they examined the extents to which children and adolescents met nutritional recommendations (54–56). An explanation for these sex-related differences could be an over-all higher need for nutrients and energy intake in males (57).

#### 4.3.2 Socioeconomic status (SES)

We found that SES was negatively associated with the consumption of the *“junk food fg”* and *“sandwiches fg”* but positively associated with the *“snacks fg.”* While the *“junk food fg”* is clearly part of an unhealthy diet, the foods in the *“snacks fg”* and *“sandwiches fg”* included both more and less healthy options.

As the frequent consumption of junk food is a strong indicator of an overall unhealthy diet, our study supports Niven et al. (58), who also noted a strong link between poor eating habits and lower SES in Australian children between the ages of 12 and 17 (58). The IDEFICS study found a negative association between SES and highly processed food (containing junk food, sweets, and snacks) consumption in seven out of eight European regions. They also found a negative association between SES and a dietary pattern dominated by sandwiches in Hungarian children. Their sandwich pattern mainly included various types of bread, cold cuts, cheese, butter, and margarine, a pattern that was well-aligned with our rather unhealthy *“sandwiches fg”* (59).

The link between higher SES and a healthier diet may be attributed to higher SES families having better knowledge and a better understanding of the importance of healthy eating. Such families are also more inclined to educate themselves about this topic (60, 61). Financial resources also play a significant role. As Biesalski noted in 2021, it is challenging to provide a healthy diet based on the financial resources available to families receiving unemployment benefits (62). Moreover, the overall food options in disadvantaged neighborhoods seem to make it more challenging to maintain a healthy diet. These areas offer fewer fruits and vegetables, while fast food and convenience stores featuring energy-dense, nutrient-poor foods are more prevalent. Additionally, advertising in these neighborhoods promotes unhealthy foods, influencing preferences and product choices (61).

Interestingly, while high SES was linked to a higher likelihood of following the *“balanced dp,”* the effect was only significant when comparing medium and high SES. Children with low SES were more likely to follow this dietary pattern than children with medium SES, but the effect was small and reached significance only marginally. Given the existing literature, the inverse association between low and medium SES would have been expected. One possible explanation for this surprising finding could be the sample size distribution in our SES groups: the medium SES group comprised 60% of the sample, whereas the low and high SES groups each represented around 20%. Furthermore, this result may indicate that the medium social status represents a transitional form between low and high social status, where the advantages of the higher social status are not present, but where similar challenges to families with low SES can be found. For example, time constraints due to work, as well as financial worries, can limit the ability to prepare healthy meals and thus lead to unhealthier choices (48, 63). At the same time, there are prevention and intervention programs aimed primarily at children from socially disadvantaged families and therefore lead to an advantage over children with a medium social status. One example of this in Germany is the education package, which enables socially disadvantaged children to participate in school and kindergarten lunches, among other things (64).

Results of the associations between snacking and SES are mixed (65). Research has found that higher SES is usually associated with healthier eating habits (58, 59), a trend that might explain our results if the healthier components of snacks, such as nuts and seeds, are considered.

#### 4.3.3 Weight groups

Consistent with Chen et al. (66), we identified a positive association between the frequent consumption of the *“meat and carbohydrates fg”* and higher weight groups. However, in a recent meta-analysis, that study was the main contributor to the significant link between high meat consumption and obesity. When that study was excluded, the statistical significance vanished (67). In our study, the *“meat and carbohydrates fg”* encompassed various types of meat along with commonly served side dishes such as pasta and rice. Thus, our results do not solely reflect the relationship between meat consumption and weight status and should be interpreted cautiously. It is therefore possible that children in our higher weight groups do not consume meat more often, but rather more carbohydrate-rich side dishes, which means that it is not possible to draw precise conclusions about their particular meat consumption. Furthermore, this combination of foods in a group can conceal dietary patterns with a particularly low or high meat intake. However, the frequency of meat consumption correlates with the consumption of side dishes.

Children with overweight or obesity tended to consume the *“vegetables fg”* more frequently but were less likely to consume the *“snacks fg”* and *“sweet bread fg.”* The impact of fruit and vegetable consumption on weight in this age group remains unclear, as noted by Ledoux et al. (68) in their systematic review. An Australian study similarly concluded that increased vegetable consumption was not correlated with lower mean BMI z-scores (45). Our findings regarding the consumption frequencies of the *“snacks fg”* and *“sweet bread fg”* are aligned with previous research (67, 69). Possible explanations include the greater satiety provided by sweet pastries compared with sugary beverages and candies (67), or stricter control by parents over nutritionally low-density foods in children with overweight (69). Otherwise, the correlation could indicate that children with overweight do not have lower quality diets than children with normal weight. This supports our previous knowledge that obesity is a multifactorial disease and that many factors, not just diet, have an influence. However, the social-desirability bias of self-reported food consumption should also be considered when interpreting these findings (70).

The *“mixed 1 dp”* and *“mixed 2 dp”* were associated with higher weight groups. In addition to frequent *“meat and carbohydrates fg”* consumption, *“mixed 1 dp”* was characterized by the highest consumption of the *“junk food fg.”* A recent systematic review highlighted the potential risk of high fast-food intake contributing to childhood and adolescent overweight (67), although a review from 2020 suggested inconsistent findings across studies regarding the direct impact of frequent fast-food consumption on obesity risk (71). When comparing the consumption frequencies of *“mixed 1 dp”* and *“mixed 2 dp,”* we found that *“mixed 1 dp”* tends to be the healthier dietary pattern. Children with overweight were linked to *“mixed 1 dp,”* while those with obesity were associated with *“mixed 2 dp.”* This suggests that as BMI increases, children may eat less healthily. Although there is a trend showing that children with higher BMI are less likely to follow the healthiest dietary pattern, the result was not significant. It is important to note that even our healthiest pattern is not an entirely optimal diet. In conclusion, a healthier diet is generally associated with lower body weight, but it cannot be considered the sole cause of obesity.

Notably, in our study, we observed very low frequencies for the consumption of the *“junk food fg”* across all dietary patterns, suggesting that previous interventions or educational programs may effectively discourage its consumption in children and adolescents. In Germany, for example, there are quality standards for catering in kindergartens (72) and schools (73), which are based on scientific guidelines from the German Nutrition Society and are intended to ensure an optimal, healthy diet for children and young people. Additionally, families participating in long-term studies such as ours are usually more interested in health education than the general population (74, 75) and again, the social-desirability bias could have influenced responses.

It was also noticeable that children with overweight and obesity were less likely to belong to the group with lower consumption frequencies but were more likely to belong to groups with higher consumption frequencies, indicating that more frequent food consumption of any kind is associated with overweight and obesity.

In our results often only the group of children with obesity was able to achieve significant results. We must highlight that due to the inclusion of our obesity cohort, we included a much higher number of children with obesity than with overweight and this has an impact on our results through group sizes. However, the trend and direction of the results was nearly always the same for both weight groups. In most cases, the effects were relatively similar for the OW and OB groups. Only in the association with the “mixed 1 dp” the effect in the OW group stand out. This could most likely be attributed to the different numbers of cases. Furthermore, a high reporting bias has been reported for subjects affected by overweight and obesity, which must also be considered as a cause here (76). This could also explain the divergence in trends when comparing the association between weight groups and the “fruits fg.”

### 4.4 Strengths and limitations

Our study examined the dietary patterns of both healthy and obese children and adolescents. By including the obesity cohort in our study, we were able to compare the diets of children with normal weight with the diets of children with obesity. In addition, even though most of the children in the SES groups were in the medium SES group, we still had considerable proportions of children in the other SES groups, especially in the low SES group, compared with other studies.

However, we used a questionnaire-based assessment of food intake, a practice that can lead to limitations, such as overreporting (76, 77), limited memory about past food intake, report bias from parents about the meals their children have outside the home, lack of concentration and motivation, as well as social desirability (70, 78, 79). Moreover, we could ask about only a limited number of different foods. However, various studies have revealed good validity and reliability compared with other methods for recording child nutrition (78, 79).

## 5 Conclusion

Our study on children’s and adolescents’ diets in the LIFE Child cohort revealed nine food groups and six distinct dietary patterns. Age, SES, and weight groups were significantly associated with these patterns or food groups, sex was associated only with food groups. In general, older children consumed fewer healthier foods, suggesting a decline in healthy eating habits as children age. Higher SES was associated with healthier eating habits, whereas lower SES was correlated with unhealthy choices. Kids with overweight and obesity reported a higher frequency of meat and carbohydrates, sandwich, and vegetable consumption but a lower consumption of snacks and sweet bread, trends that may reflect weight-related reporting bias, at least partly. Our findings suggest that age and SES are key factors that are related to children’s dietary choices, underscoring the need for age-specific, targeted nutrition education and interventions that address these social and behavioral determinants to promote healthier eating habits in youth. Children should be introduced to healthy eating and lifestyles early on to foster lifelong habits. This can be done through age-appropriate education starting in kindergarten and integrated into school curricula. Parents, as role models and primary caregivers, also need to be made more aware of the importance of healthy habits. In socioeconomically disadvantaged neighborhoods, financial support for low-income families and better access to healthy food should be prioritized. Measures like a sugar tax, reduced prices for healthy foods, and clear food labeling could help make healthier choices more accessible. Social media can be used to promote healthy eating to young people, while advertising for unhealthy foods should be restricted. Pediatricians should regularly educate both parents and children about healthy diets, with early interventions when necessary.

The results of the LIFE Child study on nutrition, such as those of the school nutrition study (43), are incorporated into Leipzig’s nutrition strategy (80), which aims to provide access to healthy, regionally produced food for all citizens, to offer more regional, seasonal and organic food and plant-based proteins in the city’s communal catering and to strengthen regional value chains in accordance with the United Nations Sustainable Development Goals (81).

Further research should be carried out to detect the complex interrelationships and their interactions. More factors that contribute to overweight and obesity should be considered so that concrete prevention and intervention programs can be developed on this basis.

## Data Availability

The datasets presented in this article are not readily available due to ethical regulations, the data on which this study are based cannot be made publicly available. The written consent for the study provided by the study participants does not include the publication of the data. Requests to access the datasets should be directed to the committee on data use and access of LIFE Child, dm@life.uni-leipzig.de.
