# Supplementary material for: Age, socioeconomic status, and weight status as determinants of dietary patterns among German youth: findings from the LIFE child study
Source: Front Nutr. 2025 Apr 30;12:1578176. doi: 10.3389/fnut.2025.1578176 (PMC12075125; doi:10.3389/fnut.2025.1578176)
Supplement: Supplementary file 1 [file Data_Sheet_1.zip › Supplementary Material.pdf]

Supplementary Material

1 Supplementary Figures and Tables

1.1 Supplementary Figures

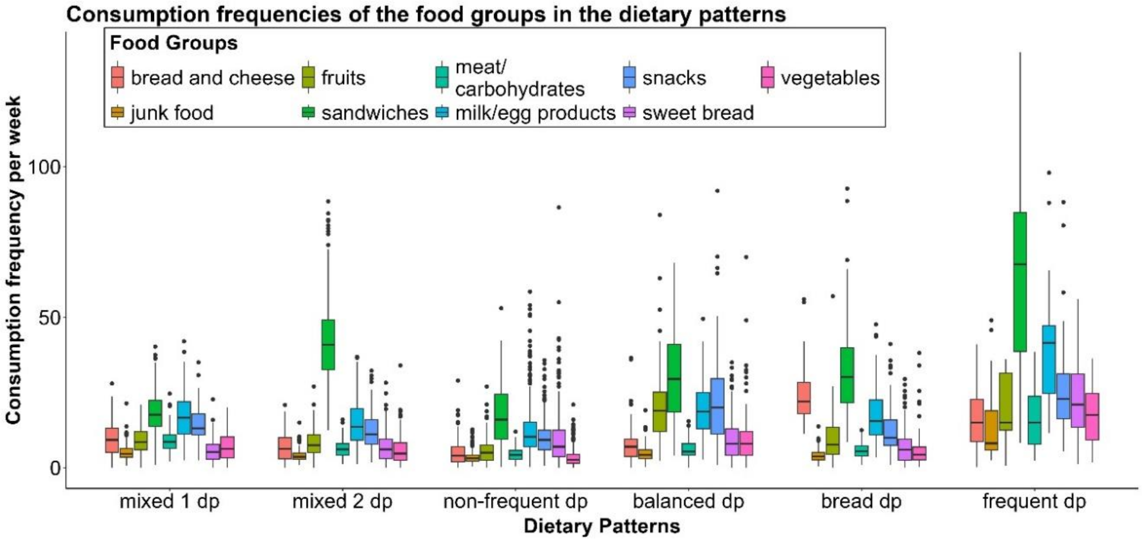

Supplementary Figure 1. Boxplot showing weekly consumption of the nine food groups in the six dietary patterns: 1: mixed 1 dp, 2: mixed 2 dp, 3: non-frequent dp, 4: balanced dp, 5: bread dp, 6: frequent dp. dp: dietary pattern.

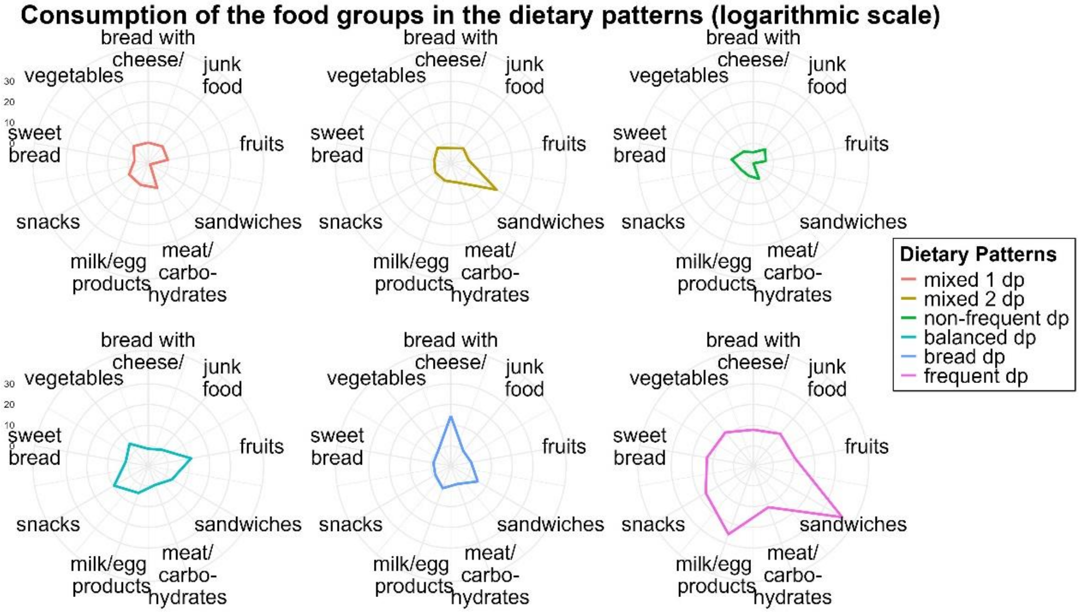

Supplementary Figure 2. Polar plot showing weekly consumption of the nine food groups (given in SDS-values) in the six dietary patterns: 1: mixed 1 dp, 2: mixed 2 dp, 3: non-frequent dp, 4: balanced dp, 5: bread dp, 6: frequent dp. dp: dietary pattern.

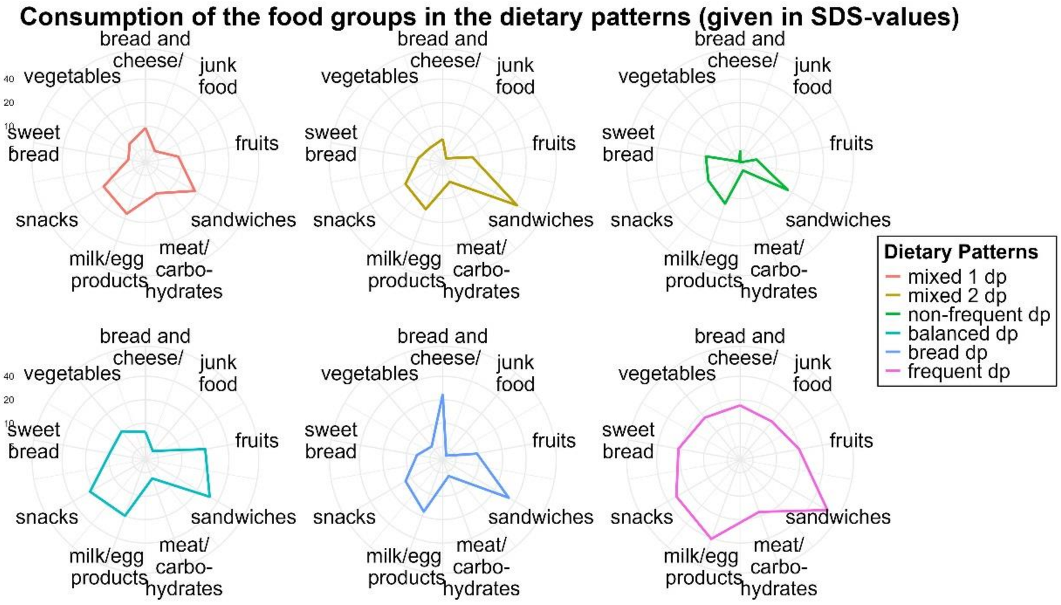

Supplementary Figure 3. Polar plot showing the weekly consumption of the nine food groups in the six dietary patterns on a logarithmic scale: 1: mixed 1 dp, 2: mixed 2 dp, 3: non-frequent dp, 4: balanced dp, 5: bread dp, 6: frequent dp. dp: dietary pattern.

1.2 Supplementary Tables

Supplementary Table 1. Associations of food groups with age, sex, SES, and weight groups. Level of significance: *p < 0.05; **p < 0.01; ***p < 0.001. SES: socioeconomic status; OB: obese; OW: overweight; NW: normal weight.

	**β**	**CI (95%)**	**p-value**
**Fruits**			
**Age**	-0.39	-0.58 – (-0.20)	< 0.001***
**Sex** (reference female)	0.65	-0.56 – 1.86	0.290
**SES**			
Medium vs. Low	0.34	-1.06 – 1.74	0.630
High vs. Low	1.01	-0.81 – 2.83	0.275
**Weight groups**			
OW vs. NW	-0.14	-1.91 – 1.62	0.874
OB vs. NW	0.55	-0.75 – 1.85	0.404
**Vegetables**			
**Age**	-0.17	-0.32 – (-0.03)	0.020*
**Sex** (reference female)	-0.51	-1.45 – 0.44	0.291
**SES**			
Medium vs. Low	-0.72	-1.76 – 0.32	0.175
High vs. Low	-0.91	-2.25 – 0.44	0.188
**Weight groups**			
OW vs. NW	0.32	-1.01 – 1.64	0.639
OB vs. NW	1.35	0.36 – 2.33	0.004**
**Meat and carbohydrates**			
**Age**	-0.11	-0.20 – (-0.01)	0.025*
**Sex** (reference female)	0.87	0.27 – 1.46	0.004**
**SES**			
Medium vs. Low	-0.44	-1.08 – 0.21	0.183
High vs. Low	-0.55	-1.38 – 0.29	0.202
**Weight groups**			
OW vs. NW	0.68	-0.14 – 1.50	0.104
OB vs. NW	0.96	0.33 – 1.58	0.003**
**Junk food**			
**Age**	-0.09	-0.17 – (-0.02)	0.017*
**Sex** (reference female)	0.51	0.04 – 0.99	0.034*
**SES**			
Medium vs. Low	-0.93	-1.45 – (-0.42)	< 0.001***
High vs. Low	-0.95	-1.62 – (-0.29)	0.005**
**Weight groups**			
OW vs. NW	0.004	-0.71 – 0.72	0.992
OB vs. NW	0.90	-0.44 – 0.62	0.74

**Snacks**			
**Age**	-0.14	-0.37 – 0.08	0.204
**Sex** (reference female)	0.88	-0.49 – 2.25	0.209
**SES**			
Medium vs. Low	1.76	0.23 – 2.39	0.024*
High vs. Low	2.69	0.74 – 4.65	0.007**
**Weight groups**			
OW vs. NW	-2.78	-4.81 – (-0.75)	0.007**
OB vs. NW	-3.42	-4.85 – (-1.99)	< 0.001***
**Milk/ egg products**			
**Age**	-0.79	-1.03 – (-0.56)	< 0.001***
**Sex** (reference female)	2.17	0.58 – 3.75	0.008**
**SES**			
Medium vs. Low	-1.15	-2.85 – 0.55	0.183
High vs. Low	-1.69	-3.92 – 0.55	0.138
**Weight groups**			
OW vs. NW	-0.58	-2.74 – 1.59	0.602
OB vs. NW	-0.25	-1.91 – 1,41	0.771
**Sweet bread**			
**Age**	-0.10	-0.28 – 0.09	0.303
**Sex** (reference female)	0.64	-0.49 – 1.76	0.266
**SES**			
Medium vs. Low	-0.66	-1.91 – 0.59	0.300
High vs. Low	-0.83	-2.41 – 0.76	0.307
**Weight groups**			
OW vs. NW	-2.23	-3.90 – (-0.57)	0.008**
OB vs. NW	-2.36	-3.53 – (-1.18)	<0.001***
**Sandwiches**			
**Age**	-0.65	-1.06 – (-0.24)	0.002**
**Sex** (reference female)	-0.36	-3.05 – 2.33	0.791
**SES**			
Medium vs. Low	-4.50	-7.42 – (-1.57)	0.003**
High vs. Low	-7.86	-11.6 – (-4.08)	< 0.001***
**Weight groups**			
OW vs. NW	2.74	-0.98 – 6.47	0.149
OB vs. NW	5.76	2.98 – 8.54	< 0.001***
**Bread and cheese**			
**Age**	-0.13	-0.33 – 0.07	0.203
**Sex** (reference female)	-0.62	-1.88 – 0.64	0.332
**SES**			
Medium vs. Low	-0.44	-1.89 – 1.00	0.547
High vs. Low	-0.02	-1.88 – 1.83	0.979
**Weight groups**			
OW vs. NW	0.91	-0.91 – 2.73	0.329
OB vs. NW	1.33	0.0005 – 2.65	0.050

Supplementary Table 2. Associations between dietary patterns and age, sex, SES, and weight groups. Level of significance: *p < 0.05; **p < 0.01; ***p < 0.001. SES: socioeconomic status; OB: obese; OW: overweight; NW: normal weight.

	**OR**	**CI (95%)**	**p-value**
**Mixed 1 dietary pattern**			
**Age**	0.95	0.82 – 1.09	0.454
**Sex** (reference female)	1.34	0.71 – 2.54	0.362
**SES**			
Medium vs. Low	1.23	0.57 – 2.68	0.598
High vs. Low	2.90	1.13 – 7.44	0.027*
High vs. Medium	2.35	1.16 – 4.77	0.018*
**Weight groups**			
OW vs. NW	4.14	1.05 – 16.3	0.042*
OB vs. NW	1.47	0.48 – 4.51	0.504
**Mixed 2 dietary pattern**			
**Age**	0.92	0.86 – 0.98	0.014*
**Sex** (reference female)	1.03	0.71 – 1.50	0.863
**SES**			
Medium vs. Low	0.77	0.48 – 1.22	0.270
High vs. Low	0.44	0.24 – 0.81	0.008**
High vs. Medium	0.57	0.35 – 0.95	0.030*
**Weight groups**			
OW vs. NW	1.49	0.83 – 2.70	0.186
OB vs. NW	2.14	1.45 – 3.15	< 0.001***
**Non-frequent dietary pattern**			
**Age**	1.16	1.08 – 1.25	< 0.001***
**Sex** (reference female)	0.73	0.48 – 1.12	0.150
**SES**			
Medium vs. Low	1.20	0.71 – 2.03	0.502
High vs. Low	1.21	0.63 – 2.33	0.564
High vs. Medium	1.01	0.61 – 1.69	0.964
**Weight groups**			
OW vs. NW	0.56	0.30 – 1.06	0.075
OB vs. NW	0.41	0.26 – 0.64	< 0.001***
**Balanced dietary pattern**			
**Age**	0.85	0.72 – 1.00	0.044*
**Sex** (reference female)	1.68	0.57 – 4.92	0.346
**SES**			
Medium vs. Low	0.85	0.70 – 1.03	0.049*
High vs. Low	1.14	0.90 – 1.43	0.195
High vs. Medium	1.33	1.10 – 1.62	< 0.001***
**Weight groups**			
OW vs. NW	0.32	0.07 – 1.52	0.150
OB vs. NW	0.60	0.19 – 1.89	0.379

**Bread dietary pattern**			
**Age**	0.97	0.89 – 1.07	0.588
**Sex** (reference female)	0.66	0.37 – 1.15	0.141
**SES**			
Medium vs. Low	1.08	0.55 – 2.12	0.831
High vs. Low	0.88	0.38 – 2.07	0.772
High vs. Medium	0.82	0.42 – 1.61	0.562
**Weight groups**			
OW vs. NW	1.76	0.80 – 3.90	0.161
OB vs. NW	1.98	1.14 – 3.44	0.015*
